# Sodium butyrate-induced DAPK-mediated apoptosis in human gastric cancer cells

**DOI:** 10.3892/or.2011.1585

**Published:** 2011-12-08

**Authors:** HYUNSOO SHIN, YEO SONG LEE, YONG CHAN LEE

**Affiliations:** 1 Department of Internal Medicine, Institute of Gastroenterology, Yonsei University College of Medicine, Seoul, Republic of Korea; 2 Brain Korea 21 Project for Medical Science, Yonsei University College of Medicine, Seoul, Republic of Korea

**Keywords:** histone deacetylase inhibitor, sodium butyrate, death-associated protein kinase

## Abstract

Epigenetic mechanisms of histone acetylation/deacetylation play an important role in the regulation of gene expression associated with the cell cycle and apoptosis. Recently, sodium butyrate, a histone deacetylase (HDAC) inhibitor, has been shown to exhibit anticancer effects via differentiation and apoptosis of cancer cells. Sodium butyrate may be a potential anticancer chemotherapeutic drug; however, the precise mechanism underlying the anticancer effects of sodium butyrate has not been clearly elucidated. In the present study, we investigated the role of death-associated protein kinase (DAPK) on the apoptosis of human gastric cancer cells induced by sodium butyrate. We observed that sodium butyrate induced apoptosis in human gastric cancer cells. Treatment with the HDAC inhibitor sodium butyrate increased the expression of *caspase-3* and *DAPK1/2* genes but decreased the expression of *Bcl-2* in human gastric cancer cells. The expression of *DAPK3*, *p53* and *p21* were not altered by sodium butyrate treatment. Analysis of the general expression patterns revealed that sodium butyrate increased the expression of *DAPK1/2* but decreased the expression of FAK and induced changes in the proliferation of apoptosis-related genes in human gastric cancer cells. These data suggest that *DAPK* expression prompts apoptosis by reducing the FAK protein level in sodium butyrate-induced apoptosis of human gastric cancer cells.

## Introduction

Histone deacetylase (HDAC) inhibitors represent a structurally diverse group of compounds that inhibit the deacetylation of histones, permitting the chromatin scaffolding to assume a more relaxed, open conformation, which generally promotes gene transcription. HDAC inhibitors induce apoptosis in cancer cells through multiple mechanisms, and thus are emerging as a promising new therapeutic tool for the treatment of a variety of human cancers (7). Sodium butyrate (NaB), a short chain fatty acid, occurs naturally in the body and is synthesized through the acetyl-CoA-dependent catabolic oxidation of long chain saturated fatty acids (1,2). Sodium butyrate, acting as an HDAC inhibitor, is known to exhibit anticancer effects via differentiation of carcinoma cells (3–6). NaB is a novel chemotherapeutic agent as it is able to activate a variety of anticancer mechanisms, including cell cycle arrest and cellular differentiation (8–10). Sodium butyrate induces apoptosis, decreases Bcl-2 transcription (11), increases TNF-related apoptosis-inducing ligand receptor 2 gene transcription to accelerate the death-inducing signaling complex formation, activates caspase, and inhibits the mitochondrial membrane potential of cancer cells (12). These results suggest that NaB may represent a potential new class of anticancer agents with low toxicity; however, the mechanisms of action are not fully understood.

DAPK induces programmed cell death through various signaling pathways. FAK participates in signaling pathways involved in adhesion between cells and the extracellular matrix, including proteins such as fibronectin, laminin, actin, and fodrin. FAK is a survival protein that suppresses apoptosis and maintains cell suspended growth (26). In this study, we attempted to elucidate NaB-induced death-associated protein kinase expression and its association with human gastric cancer cell apoptosis.

## Materials and methods

### Cell culture and treatments

A total of nine human gastric cancer cell lines (AGS, Kato III, MKN28, MKN45, MKN74, NCI-N87, SNU1, SNU16 and SNU638) were obtained from the Korean Cell Line Bank (Korea) and the American Type Culture Collection (Manassas, VA, USA). Cells were cultured in RPMI-1640 medium (Hyclone Laboratories, Inc., USA) containing 10% fetal bovine serum (Hyclone) and 1% penicillin streptomycin sulfate (Hyclone) at 5% CO_2_, 37°C, and 95% humidity. Sodium butyrate, 5′-Aza-2-deoxycytidine (5′-Aza-dC), and trichostatin A were purchased from Sigma-Aldrich (St. Louis, MO, USA). Working concentrations were as follows: sodium butyrate (NaB), 2 μM; 5-Aza-dC, 2 μM; and trichostatin A, 200 nM.

### RNA isolation and reverse transcription polymerase chain reaction

Total RNA was prepared using TRIzol reagent (Invitrogen). The RNA was reverse transcribed using oligo (dT) primers and SuperscriptT™ II reverse transcriptase (Invitrogen, Carlsbad, CA, USA). Primers used for PCR amplification of this cDNA were caspase-3: forward, 5′-GGC ATT GAG ACA GAC AGT GGT G-3′ and reverse, 5′-GCA CAA AGC GAC TGG ATG AAC C-3′; Bcl-2: forward, 5′-GAG TAC CTG AAC CGG CAC CT-3′ and reverse, 5′-CAG GGT GAT GCA AGC TCC CA-3′; DAPK1: forward, 5′-TCT ACC AGC CAC GGG ACT TC-3′ and reverse, 5′-GCT GGC CTG TGA GTA GAC GT-3′; DAPK2: forward, 5′-GCA TCG TGT CCC TGT GCA AC-3′ and reverse, 5′-GCT TTC CTC CTG GCG ATG TC-3′; DAPK3: forward, 5′-CCC AAC CCA CGA ATC AAG CTC-3′ and reverse, 5′-GCT GAG ATG TTG GTG AGC GTC-3′; p21: forward, 5′-GTA CCC TTG TGC CTC GCT CA-3′ and reverse, 5′-CCG GCG TTT GGA GTG GTA GA-3′; p53: forward, 5′-AGC GAT GGT CTG GCC CCT CCT-3′ and reverse, 5′-CTC AGG CGG CTC ATA GGG CAC-3′; and β-actin: forward, 5′-TTG CCG ACA GGA TGC AGA AG-3′ and reverse, 5′-AGG TGG ACA GCG AGG CCA GG-3′. PCR reactions were performed with the PCR Maxi kit (Intron, Sungnam, Korea). PCR reactions in the linear range of amplification were analyzed by agarose gel electrophoresis and quantified by densitometry, if needed.

### Western blot analysis

Prepared cells were harvested after washing with PBS. Collected cells were lysed with buffer [50 mM Tris-Cl (pH 7.5), 150 mM NaCl, 1 mM EDTA (pH 8.0), 1% Triton X-100, 1 mM PMSF, 1 mM Na_3_VO_4_, and protease inhibitor cocktail (Roche Molecular Biochemicals, Indianapolis, IN, USA)]. Fractionation was performed by sequential extraction of cytosolic and nuclear proteins in non-ionic detergent for analysis of β-catenin. The same amount of protein was boiled at 95°C after adding SDS sample buffer [62.5 mM Tris-Cl (pH 6.8), 2% sodium dodecyl sulfate, 10% glycerol, β-mercaptoethanol, and 0.002% bromophenol blue]. Samples were loaded on 8% SDS-PAGE gels for DAPK1 and FAK and on 10% SDS-PAGE gels for DAPK2 analyses and transferred to PVDF membranes (Amersham Biosciences, Pisctaway, NJ, USA). Anti-DAPK1 (Santa Cruz Biotechnology, Santa Cruz, CA, USA), Anti-DAPK2 (Santa Cruz Biotechnology), and anti-FAK (Santa Cruz Biotechnology) were used as the primary labeling antibodies, and the appropriate horseradish peroxidase-conjugated antibodies (Santa Cruz Biotechnology) were used as secondary antibodies. An enhanced chemiluminescence detection system (ECL-Plus, Intron, Seoul, Korea) was used for detection according to the manufacturer’s protocol.

### Immunofluorescence microscopy

Human gastric cancer cells were cultured on chamber slides and then washed with phosphate-buffered saline (PBS) and fixed with 10% formaldehyde. They were then incubated with anti-DAPK1, anti-DAPK2, and anti-FAK antibodies and stained with anti-goat IgG-FITC and anti-rabbit IgG-FITC antibodies (all from Santa Cruz Biotechnology). Cells were visualized using a Zeiss LSM 510 confocal laser-scanning microscope (Carl Zeiss, USA).

### Flow cytometry

Human gastric cancer cells were treated with 2 μM NaB, 2 μM 5′-Aza-dC for 48 h, replacing the drug and medium every 24 h. Prepared cells were harvested after washing with PBS. Collected cells were lysed with 1X binding buffer (BD Biosciences, USA). One hundred microliters of the lysate was transferred to a 5 ml culture tube and treated with FITC Annexin-V and PI (BD Biosciences) for 15 min at 25°C in the dark. The cell cycle distribution was determined using a FACScan flow cytometer (Becton-Dickinson, Mountain View, CA, USA) and 10,000 cells were analyzed with the MultiCycle software package (Phoenix Flow Systems, San Diego, CA, USA).

### Cell proliferation assay

Human gastric cancer cells were seeded in 96-well plates. Cells were treated with 2 μM NaB, 2 μM 5′-Aza-dC and 200 nM trichostatin A for 48 h, replacing the drug and medium every 24 h. The colorimetric MTS (Promega) assay was used to measure cell numbers at 48 h, according to the manufacturer’s manual. Experiments were performed in triplicate.

## Results

### Sodium butyrate inhibited cell proliferation

**Figure 1:**
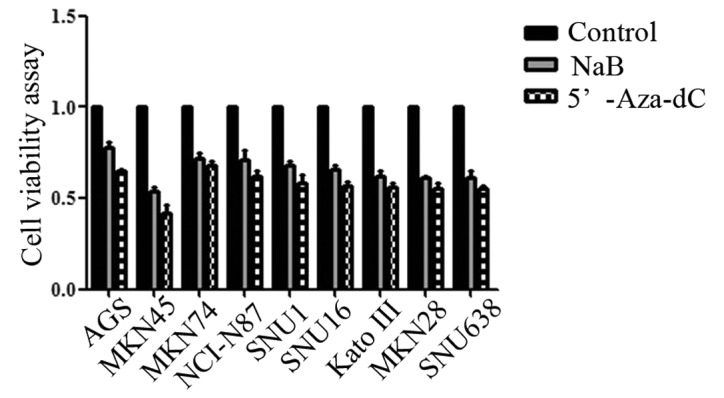
Treatment with NaB inhibits human gastric cancer cell proliferation. Cells were seeded in 96-well plates. The colorimetric MTS assays were used to measure cell numbers at 48 h. Experiments were performed in triplicates. NaB, sodium butyrate.

We examined the effects of NaB on human gastric cancer cell proliferation. After 48 h of NaB treatment, cell viability was reduced from 0.58 to 0.41 in AGS, from 0.40 to 0.22 in MKN45, from 0.63 to 0.34 in MKN74, from 0.66 to 0.39 in NCI-N87, from 1.2 to 0.63 in SNU1, from 0.44 to 0.21 in SNU16, from 0.42 to 0.19 in Kato III, from 0.45 to 0.19 in MKN28, and from 0.99 to 0.45 in SNU638 (all P<0.05) as determined by the cell proliferation assay (Fig. 1).

### Sodium butyrate induced apoptosis of human gastric cancer cells

**Figure 2:**
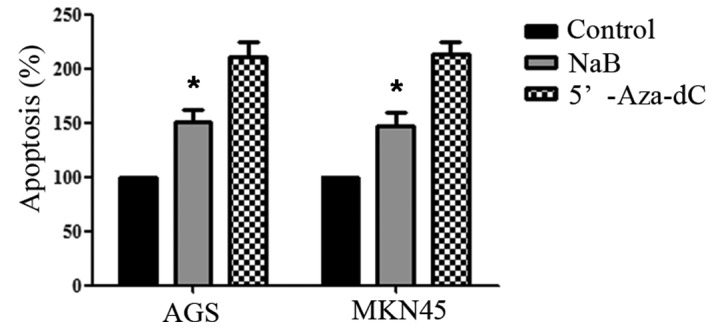
Apoptosis induced by NaB. Cells were harvested and analysed 48 h after treatment with sodium butyrate. Apoptotic cells are indicated as the sub-G1 fraction, and percentages are shown on the top. NaB, sodium butyrate.

Cell apoptosis was determined by counting sub-G1 phase cells with flow cytometry analysis. After 48 h of NaB treatment, cell apoptosis significantly increased from 0.05 to 7.2% in AGS and from 0.07 to 5.83% in MKN45 (both P<0.05). These findings suggested that NaB induced cell apoptosis (Fig. 2).

### Effect of sodium butyrate on apoptosis-regulated protein expression

**Figure 3:**
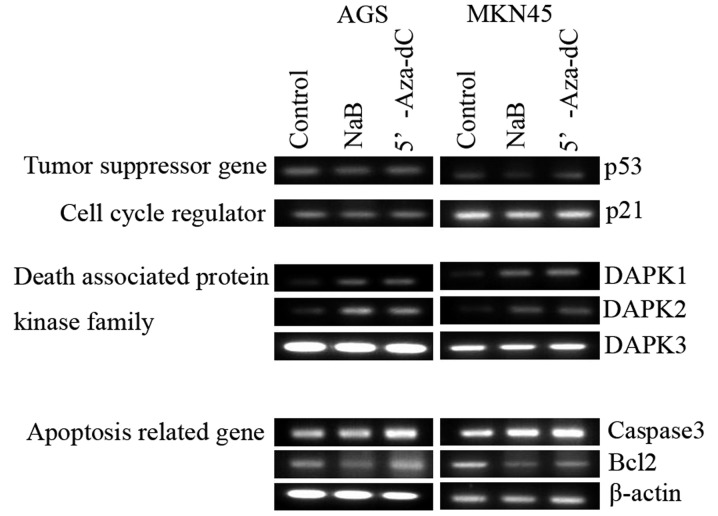
Effect of NaB on gene expression by RT-PCR. *Caspase-3, Bcl-2*, *DAPK1, DAPK2, DAPK3*, *p53* and *p21* expression in the indicated human gastric cancer cell lines, with treatment of NaB, 5,-Aza-dC. RT-PCR for β-actin was carried out for all samples for control. NaB, sodium butyrate.

Sodium butyrate increased the expression of *caspase-3*, *DAPK1*, and *DAPK2* but decreased the expression of *Bcl-2* in both AGS and MKN45 cells. The expression of *DAPK3*, p*53* and *p21* genes was not altered by NaB treatment (Fig. 3). These finding suggests that NaB induces *DAPK1/2* expression in human gastric cancer cells.

### Sodium butyrate increased DAPKs expression and decreased the expression of FAK in human gastric cancer cells

**Figure 4:**
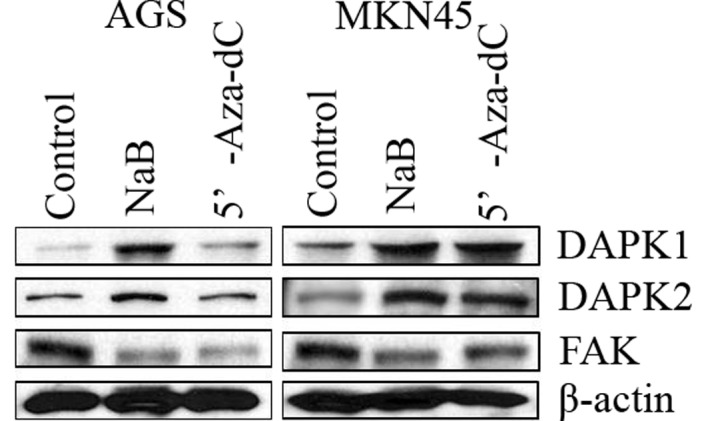
Effect of NaB on the protein level of DAPK1, DAPK2, FAK in human gastric cancer cell lines. The loading and transfer of equal amounts of protein were confirmed by immunodetection of β-actin. NaB, sodium butyrate.

To confirm DAPKs expression induced by NaB treatment, Western blot analysis was performed 48 h after treatment with NaB. Expression of DAPK1/2 was increased, while that of FAK was decreased in human gastric cancer cells (Fig. 4). These finding suggest that NaB induced the expression of DAPK1/2, leading to decreased protein levels of FAK, prompting cell apoptosis.

### Increased DAPKs expression and decreased FAKs expression induced by sodium butyrate treatment demonstrated by immunofluorescence

**Figure 5:**
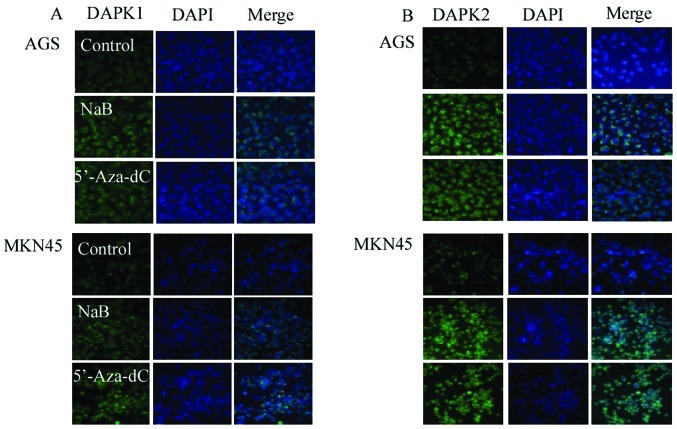
Increased DAPK1/2 expression by butyrate treatment in human gastric cell lines. Immunofluorescence assay of the intracellular distribution DAPK1/2 in human gastric cell lines (A and B). Cells were stained with anti-DAPK1/2 antibody (green). Nuclei were visualized using 4′,6-diamidino-2-phenylindole (DAPI, blue).

**Figure 6:**
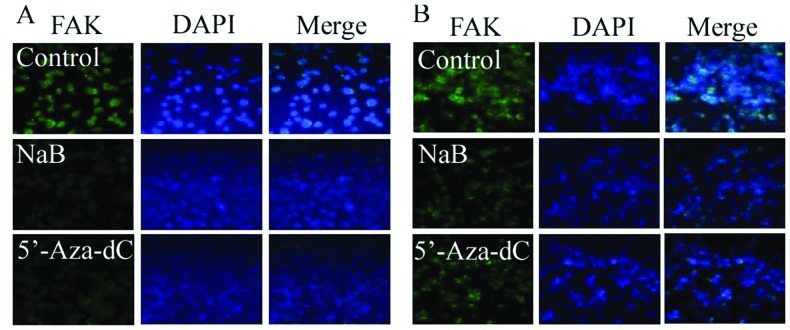
Decreased FAK expression by butyrate treatment in human gastric cell lines. Immunofluorescence assay of the intracellular distribution of FAK in human gastric cell lines. Cells were stained with anti-FAK antibody (green). Nuclei were visualized using 4′,6-diamidino-2-phenylindole (DAPI; blue) (A) AGS, (B) MKN45 cells.

To confirm DAPKs expression induced by NaB, an immunofluorescence assay was analyzed 48 h after treatment of cells with NaB. Sodium butyrate increased the expression of the DAPK1/2 protein but decreased the expression of FAK. These findings suggest that NaB induced the expression of *DAPK1/2*, leading to decreased protein levels of FAK (Figs. 5 and 6).

## Discussion

Sodium butyrate enhances the response of chemotherapeutic agents in human gastric cancer cells (13–15). Consistent with previous studies, we have confirmed that sodium butyrate can induce demethylation of the SFRP gene promoter. HDAC inhibitors are capable of inducing apoptosis and cell cycle arrest at the G1/G2 phase by altering gene expression (16–23).

In the current study, we aimed to assess sodium butyrate-induced death-associated protein kinase expression, which promotes human gastric cancer cell apoptosis. We first sought to assess the role of sodium butyrate in human gastric cancer cells using several independent approaches. Our results suggest that sodium butyrate inhibits cell proliferation and induces cell apoptosis in human gastric cancer cells.

The level of *caspase-3* mRNA expression increased in human gastric cancer cells following treatment with sodium butyrate. Although no studies support the fact that the transcription of *caspase-3* can be upregulated directly by histone acetylation, recent evidence suggests that HDAC inhibitors also enhance the acetylation of non-histone proteins (24,25). Down-regulation of *Bcl-2* was present after 48 h of exposure to sodium butyrate. These findings suggest that *caspase-3* was increased by sodium butyrate in human gastric cancer cells, but not *Bcl-2*. The *p53* gene is not involved in butyrate-induced growth inhibition of breast cancer cells (26). The current study also demonstrates that sodium butyrate did not induce *p53* or *p21* expression. Recently, it has been reported that the histone deacetylase inhibitor, TSA, could induce cells to express *DAPK* and promote cell apoptosis (24). *DAPK* expression causes tumor cells to lose sensitivity to anoikis, enabling anchor-independent survival of tumor cells (25). Our results found that *DAPK1/2*, but not *DAPK3*, expression was increased by sodium butyrate treatment in human gastric cancer cells. *DAPK1/2* is responsible for the induction of apoptosis, while *DAPK3* usually induces morphological changes in apoptosis.

FAK is involved in adhesion between cells and the extra-cellular matrix, and joins cytoskeletal proteins. FAK is a survival protein that suppresses apoptosis (23). Our experiments demonstrate that sodium butyrate induced *DAPK1/2* expression but down-regulated FAK expression in human gastric cancer cells. These findings suggest that a sodium butyrate-induced *DAPK*-dependent decrease in FAK via the caspase-dependent pathway leads to apoptosis in human gastric cancer cells. In conclusion, sodium butyrate induced *DAPK* expression in human gastric cancer cells and this expression prompted apoptosis by decreasing FAK levels.
